# Human Aquaporin 4 Gene Polymorphisms and Haplotypes Are Associated With Serum S100B Level and Negative Symptoms of Schizophrenia in a Southern Chinese Han Population

**DOI:** 10.3389/fpsyt.2018.00657

**Published:** 2018-12-11

**Authors:** Yung-Fu Wu, Huey-Kang Sytwu, For-Wey Lung

**Affiliations:** ^1^Department of Psychiatry, Beitou Branch, Tri-Service General Hospital, National Defense Medical Center, Taipei, Taiwan; ^2^Graduate Institute of Medical Science, National Defense Medical Center, Taipei, Taiwan; ^3^Department of Microbiology and Immunology, National Defense Medical Center, Taipei, Taiwan; ^4^National Health Research Institutes, Zhunan, Taiwan; ^5^Calo Psychiatric Center, Pingtung, Taiwan

**Keywords:** schizophrenia (SCZ), aquaporin 4 (AQP4), single nucleotide polymorphism (SNP), haplotype, S100 calcium-binding protein B (S100B)

## Abstract

**Background:** Aquaporin 4 (AQP4) polymorphism may influence the required dosage of antipsychotic drugs. However, the roles of AQP4 polymorphisms in the blood—brain barrier (BBB) and different neuroprotective effects need further exploration. This study aims to investigate whether the gene polymorphisms and haplotype of AQP4 are associated with serum S100 calcium-binding protein B (S100B) level and clinical symptoms in patients with schizophrenia (SCZ).

**Methods:** We recruited 190 patients with SCZ. They provided demographic data, completed relevant questionnaires, and submitted samples to test for four AQP4 tag single nucleotide polymorphisms (SNPs) and eight haplotypes. The rating scales of Positive and Negative Syndrome Scale (PANSS), Personal and Social Performance (PSP), the Global Assessment of Functioning (GAF), Clinical Global Impression (CGI) were assessed and serum S100B level were measured repeatedly during antipsychotic treatment at weeks 0 (baseline), 3, 6, and 9. Using generalized estimating equation (GEE) analyses, log-transformed S100B (logS100B) level was tested for associations with haplotype and other dependent variables.

**Results:** Discretization via the median split procedure showed that logS100B level >1.78 or ≤ 1.78 had the best discriminant validity to stratify the patients into two groups. After 9 weeks of treatment, the serum S100B level was decreased. The TAA haplotype of AQP4 SNPs was associated with increased serum S100B level (*p* = 0.006). The PANSS negative subscale (PANSS-N) (*p* = 0.001) and Clinical Global Impression–Improvement (CGI-I) (*p* = 0.003) scores had a positive association with S100B level.

**Conclusion:** Patients with the TAA haplotype of the AQP4 polymorphism are likely to have increased serum S100B level, negative symptoms and poor control of neuroinflammation. A logS100B level >1.78 may be sufficiently specific to predict a higher severity of negative symptoms. Further study including healthy controls and patients with first and recurrent episodes under selective AQP4 modulators will be necessary to explore the profound effects on the treatment of patients with SCZ and may positively influence their overall outcome.

## Background

Schizophrenia (SCZ) is a complex chronic psychiatric disorder; the clinical psychopathology involves cognition, perception, emotion, and other appearances of behavior although the presentation of these features differs across patients and over time. The lifetime prevalence of SCZ is 0.6–1.9% (1). Recent genome-wide studies suggest immune involvement in SCZ (2). Sustained inflammatory activation of microglia and astrogliosis are important mechanisms in the progression of neuroinflammation. Neuroinflammation is frequently associated with blood—brain barrier (BBB) dysfunction and a common pathological event observed in neuropsychiatric diseases. An accumulating body of evidence points to the association between neuroinflammation and SCZ (3). Increasing numbers of studies have shown that SCZ involves a chronic process of neuroinflammation in the brain (4, 5).

Aquaporin-4 (AQP4) is a water-channel protein and highly expressed in the human body primarily at the end-feet of astrocytes surrounding capillaries (6, 7). In addition, AQP4 is involved in BBB development, function, and integrity (8). Apart from its function in water homeostasis, many studies have shown possible inter-relations between AQP4 and neuroinflammation (9). This protein plays an important role in various brain pathological conditions, such as SCZ (10, 11).

S100 calcium-binding protein B (S100B) is a member of the S100 protein family and abundant in astroglial cells. The protein has therefore been considered a glial marker protein (12–14). Given its high level of expression in brain tissue, most studies of the relation between S100 and neurodegeneration have focused on S100B in particular. Increased S100B concentrations are mainly considered to be result of astroglial or BBB dysfunction. Scientific studies have mentioned that blood levels of S100B are increased in SCZ (15–18). Therefore, S100B may be useful in the development of a diagnostic biomarker signature of SCZ (19).

The existence of single nucleotide polymorphisms (SNPs) from different forms of DNA sequence variation may explain the possible genetic risk for SCZ (20). Our previous study found that AQP4 polymorphism may influence the required dosage of antipsychotic drugs (11). However, the roles of AQP4 polymorphisms in the BBB and different neuroprotective effects need further exploration. Here, we hypothesized that astroglial AQP4 would play a crucial role in the regulation of the extent of neuroinflammation and the illness severity in SCZ. We aimed to investigate whether the gene polymorphisms and haplotype of AQP4 are associated with serum S100B level and clinical symptoms in patients with SCZ, while controlling related factors.

## Methods

### Participants and Procedure

A total of 190 patients with SCZ completed the study from the psychiatric wards or outpatient departments in Taiwan. All the patients with SCZ were interviewed face-to-face by a senior psychiatrist, and fulfilled the criteria for SCZ, based on the Mini International Neuropsychiatric Interview (MINI) (21) for DSM-IV criteria and the International Classification of Diseases-10 (WHO-ICD-10). This study was approved by the Independent Ethics Committee/Institutional Review Board in Taiwan. The exclusion criteria were: abnormal value of C-reactive protein (CRP) or erythrocyte sedimentation rate (ESR); psychosis other than SCZ; intellectual disability; substance-related and addictive disorders; other neurocognitive disorders; and having undergone electroconvulsive therapy within the past 6 months. The exclusion criteria also included: active medical illnesses that could be etiologically related to the level of S100B (e.g., uncontrolled cancer, autoimmune or infectious diseases, or a cardiovascular incident within the past 6 months). All participants provided written informed consent, demographic data and completed relevant assessment of clinical symptoms and global function. Whole blood samples were obtained in the morning for testing. The EDTA-anticoagulated blood samples were collected by venipuncture for DNA extraction, tag SNPs and subsequent SNP genotyping. An additional venous blood sample was withdrawn for human serum S100B ELISA detection at the same time. Both the assessment of clinical symptoms and the detection of serum S100B level were recorded at baseline (week 0), and at 3, 6, and 9 weeks. Each recruited participant with SCZ took typical and atypical antipsychotics according to their physician's choice.

### Demographic Variables

A self-report questionnaire was used to obtain demographic information, including age, gender, educational level, marital status, military service, age at diagnosis of SCZ, smoking and family history of mental disorder.

### Isolation of DNA, SNP Selection, and Genotyping

Genomic DNA was extracted from peripheral blood leukocytes using a salting out method. Based on the HapMap data for Han Chinese in the Beijing population, tag SNPs across the entire region of the AQP4 gene were selected using the tagger algorithm (http://www.broadinstitute.org/mpg/tagger/) with a pairwise approach, an *r*^2^ cutoff of 0.8 and a minor allele frequency >0.05. A total of four tag SNPs in two distinct gene regions were retrieved: in the 3′UTR region (rs1058424, rs335929, rs3763043) and in the intronic region between exons 4 and 5 (rs335931). Tag SNP genotyping was performed with TaqMan allele-specific discrimination assays on an ABI PRISM_7700 Sequence Detection System and analyzed with the SDS software (Applied Biosystems, Foster City, CA).

### Haplotype Reconstruction

We use PHASE (22) to estimate the haplotypes from the tag SNPs. The software PHASE version 2.1.1 (http://stephenslab.uchicago.edu/phase/download.html) uses a Gibbs sampling approach in which each individual haplotypes is updated conditionally upon the current estimates of haplotypes from all other samples. Approximations to the distribution of a haplotype conditional upon a set of other haplotypes were used for the conditional distributions of the Gibbs sampler.

### Human S100B ELISA Detection

Samples of venous blood (5 ml) were withdrawn to measure the level of S100B protein. Blood samples were collected by venipuncture into tubes containing heparin. Plasma samples were centrifuged at 1,008 g for 10 min. The samples were maintained at −80°C before performing the assays. The concentration of human S100B in serum was measured with an ELISA kit. A Multiskan FC microplate photometer (Thermo Scientific) was used for reading, at 450 nm. The results were expressed in pg/ml.

### Clinical Symptoms and Function Assessment

Assessment of clinical symptoms and function involved the rating scales of Positive and Negative Syndrome Scale (PANSS), the Global Assessment of Functioning (GAF), Personal and Social Performance (PSP), Clinical Global Impression–Severity (CGI-S) and Clinical Global Impression–Improvement (CGI-I).

### Data Processing and Statistical Analyses

S100B values were log-transformed to normalize data because of their non-linear distribution. To define groups with high and low baseline levels of S100B, this variable was dichotomized by a median split. The cut-off value of the baseline log-transformed S100B (logS100B) is determined to be 1.78. To investigate the differences between groups, we genotyped patients for the risk SNPs. The Hardy–Weinberg equilibrium (HWE) of the four SNPs was calculated for the allele and genotype frequencies. A goodness-of-fit χ^2^ test was used to detect the HWE, and Pearson χ^2^ test to compare allele distribution comparison. SPSS 23.0 (SPSS Inc., Chicago, IL) was used for demographic analysis, descriptive analysis, and exploratory analysis. Logistic regression analysis was used to investigate the risk level of the tested SNPs associated with SCZ, before and after adjustment for age and gender. The generalized estimating equation (GEE), developed by Zeger and Liang in 1986 (23), was applied to handle our missing data due to absenteeism or a failure to complete the assessment on time. The primary objective of this analysis was to explore the relationship between clinical effects and serum logS100B levels. The GEE was used to analyze the independent variables of gender, age, age at onset, duration of illness, educational level, military service, haplotypes of tag SNPs, smoking history, family history of mental disorder and other outcome variables. The dependent variable was set as the logS100B level.

## Results

**Figure 1:**
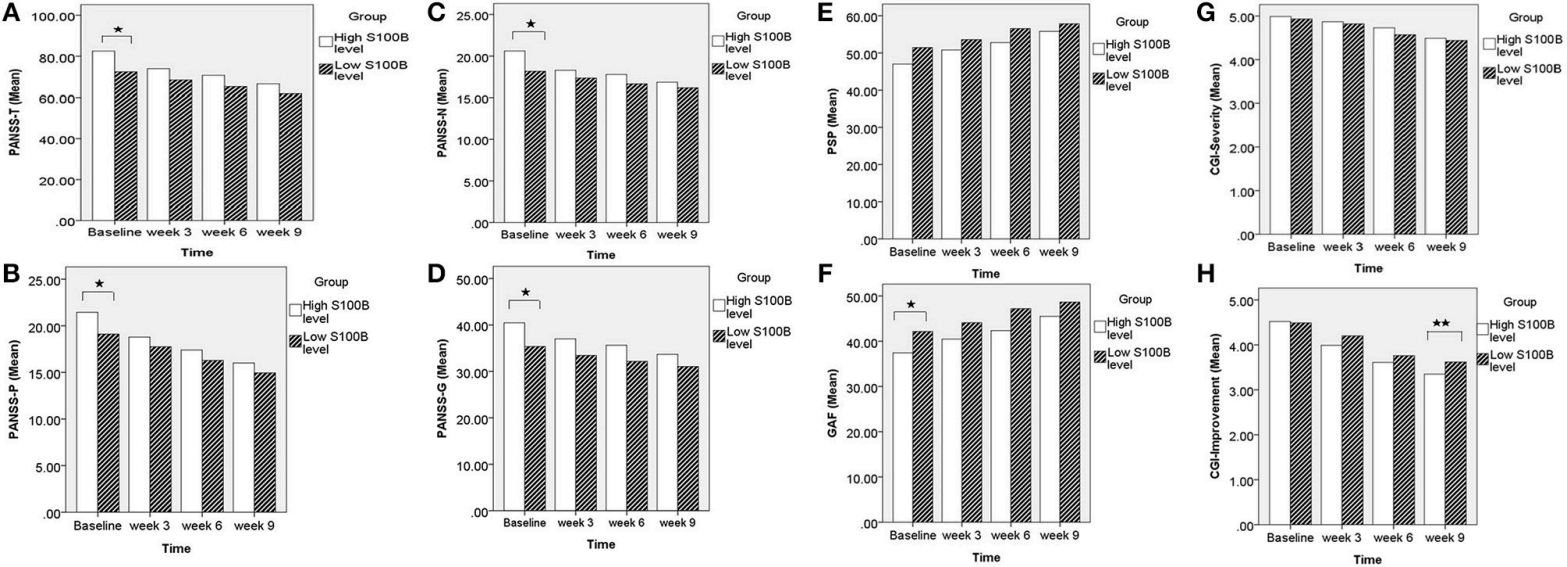
Comparing the high S100B group to the low S100B group, the significant *P*-values were observed in the **(A)** baseline PANSS total scores (*p* = 0.01) as well as the **(B)** positive (*p* = 0.035), **(C)** negative (*p* = 0.021), and **(D)** general (*p* = 0.017) subscales. Also, the *P*-values of both **(F)** baseline GAF level (*p* = 0.035) and **(H)** final CGI-I scores (*p* = 0.006) had significance. *p < 0.05. **p < 0.01.

A total of 190 patients with SCZ from the Southern Chinese Han population participated in our study. 93 of 190 patients joined our study in acute relapse and 97 of 190 patients were in stable condition. 77.5% patients were treated with 1 antipsychotic drug (monotherapy) and 22.5% patients received 2 or more different antipsychotics (polypharmacy). Table 1 shows the clinical and demographic information of the participants, and baseline statistics between groups. We recorded each participant's age, gender, educational level, smoking, marital status, family history of mental disorder, military service, age at diagnosis of SCZ, and baseline ESR and CRP levels. The *P*-values of the above variables showed no significant differences between groups. Repeated measurements of PANSS, PSP, GAF, CGI-S, CGI-I scores and S100B level were collected in a longitudinal study in which change over time is assessed. Comparing the high S100B group to the low S100B group, the significant *P*-values were observed in the baseline PANSS total scores (*p* = 0.01) as well as the positive (*p* = 0.035), negative (*p* = 0.021) and general (*p* = 0.017) subscales (Table 2). Also, the *P*-values of both baseline GAF level (*p* = 0.035) and final CGI-I scores (*p* = 0.006) had significance. Multiple bar graphs were included to show the changes of S100B, PANSS, and other outcome variables derived from different time-points between groups (Figure 1).

**Table 1:** Clinical and demographic information of the participants, and baseline statistics between groups.

	**High S100B group (*****n*** **=** **95)**		**Low S100B group (*****n*** **=** **95)**		***p*-value**
**Variables**	**Number**	**Percent (%)**	**Number**	**Percent (%)**
Male	46	48.4	52	54.7	0.468
Age distribution (years)					0.113
31–40	13	13.7	21	22.1	
41–50	33	34.7	34	35.8	
51–60	38	40.0	23	24.2	
61–70	11	11.6	17	17.9	
Educational level (years)					0.228
< 6	12	12.6	17	17.9	
7–9	32	33.7	30	31.6	
10–12	35	36.8	24	25.3	
>13	16	16.8	24	25.3	
Smoking (with)	27	28.4	38	40.0	0.126
Marriage (with)	22	23.2	32	33.7	0.147
History of mental disorder (with)	29	30.5	21	22.1	0.190
Military service (with)	23	24.2	28	29.5	0.513
	**Mean**	***SD***	**Mean**	***SD***	***p*****-value**
Age at diagnosis of SCZ (years)	25.41	9.40	25.92	8.88	0.520
Duration of illness (years)	23.60	10.67	22.72	11.19	0.139
ESR^*a*^	21.68	19.46	17.42	14.19	0.760
CRP^*b*^	0.47	0.80	0.36	0.48	0.382

a *ESR, erythrocyte sedimentation rate*;

b *CRP, C-reactive protein*.

**Table 2:** Analysis of outcome variables derived from different time-points between groups.

**Variables**	**Time**	**High S100B group**			**Low S100B group**			***p*-Value**
		***n***	**Mean**	**SE**	***n***	**Mean**	**SE**
S100B	Baseline	95	165.22	22.00	95	32.57	1.58	0.178
	W3	89	136.65	28.82	91	40.97	3.37	0.413
	W6	85	138.00	26.67	87	44.23	4.14	0.456
	W9	79	98.66	18.34	81	33.38	1.95	0.454
PANSS-T	Baseline	95	82.54	1.66	95	72.60	1.95	0.010^*^
	W3	89	74.12	1.57	91	68.51	1.51	0.529
	W6	85	70.85	1.53	87	65.28	1.37	0.252
	W9	79	66.58	1.32	81	61.80	1.28	0.175
PANSS-P	Baseline	95	21.45	0.53	95	19.11	0.81	0.035^*^
	W3	89	18.78	0.50	91	17.75	0.74	0.388
	W6	85	17.40	0.51	87	16.29	0.68	0.457
	W9	79	16.01	0.45	81	14.95	0.43	0.839
PANSS-N	Baseline	95	20.62	0.47	95	18.18	0.46	0.021^*^
	W3	89	18.30	0.42	91	17.36	0.37	0.451
	W6	85	17.79	0.36	87	16.67	0.34	0.376
	W9	79	16.87	0.37	81	16.19	0.32	0.142
PANSS-G	Baseline	95	40.46	0.91	95	35.35	1.05	0.017^*^
	W3	89	37.00	0.86	91	33.43	0.83	0.108
	W6	85	35.62	0.87	87	32.16	0.75	0.269
	W9	79	33.68	0.76	81	31.04	0.74	0.476
PSP	Baseline	95	47.01	1.07	95	51.46	1.05	0.115
	W3	89	50.80	0.92	91	53.59	0.81	0.597
	W6	85	52.80	0.93	87	56.55	0.70	0.738
	W9	79	55.81	0.90	81	57.85	0.86	0.422
GAF	Baseline	95	37.42	1.06	95	42.16	1.06	0.035^*^
	W3	89	40.48	0.98	91	44.11	0.89	0.241
	W6	85	42.35	0.96	87	47.20	0.76	0.115
	W9	79	45.51	0.99	81	48.64	0.81	0.362
CGI-S	Baseline	95	4.99	0.05	95	4.93	0.06	0.626
	W3	89	4.87	0.06	91	4.82	0.05	0.257
	W6	85	4.73	0.06	87	4.57	0.06	0.139
	W9	79	4.49	0.08	81	4.44	0.09	0.839
CGI-I	Baseline	95	4.52	0.08	95	4.49	0.08	0.305
	W3	89	3.99	0.09	91	4.20	0.08	0.316
	W6	85	3.61	0.09	87	3.76	0.09	0.547
	W9	79	3.35	0.09	81	3.62	0.10	0.006^**^

* *p < 0.05*,

** *p < 0.01*.

*S100B, S100 calcium-binding protein B; PANSS-T, total scores of the positive and negative syndrome scale; PANSS-P, positive subscale of the positive and negative syndrome scale; PANSS-N, negative subscale of the positive and negative syndrome scale; PANSS-G, general subscale of the positive and negative syndrome scale; PSP, personal and social performance; GAF, global assessment of functioning; CGI-S, clinical global impression-severity; CGI-I, clinical global impression–improvement*.

Table 3 shows the characteristics of the genotyped AQP4 among the four tag SNPs. Table 4 shows the allele and genotype frequencies for each tag SNP. The T allele of rs1058424 has the significance between two groups (*p* = 0.013). Among the four genotyped tag SNPs, rs335931 exhibited a low level of linkage disequilibrium and therefore was excluded. We chose the three other tag SNPs (rs1058424, rs335929, and rs376043) for further PHASE analysis. The Table 5 showed that the TAA haplotype was significantly different between the two groups (*p* = 0.018), as was the ACG haplotype (*p* = 0.048). Although the *P*-value of these two haplotypes was insignificant after the Bonferroni correction, the FDR adjusted *P*-value of TAA haplotype was still meaningful due to the less likely to be false positive.

**Table 3:** Characteristics of genotyped AQP4 tag SNPs.

	**rs number**	**Chromosome position**	**Distance from gene start**	**Gene position**	**Function**	**MAF**
1	rs1058424	24,435,545	3,543	3′UTR	Regulatory	A/T (0.500)
2	rs335929	24,435,587	3,585	3′UTR	Regulatory	A/C (0.447)
3	rs3763043	24,435,818	3,816	3′UTR	Regulatory	G/A (0.389)
4	rs335931	24,439,072	7,070	Intron 4–5	No-coding	G/A (0.337)

*MAF, Minor allele frequency*.

**Table 4:** Genotype and allele frequencies of AQP-4 tag SNPs between groups.

**AQP-4 tag SNPs**		**High S100B group (*n* = 95)**	**Low S100B group (*n* = 95)**	***p*-value**
**rs1058424**				
Genotype				
	TT	34	22	0.141
	TA	37	41	
	AA	24	32	
Allele				
	T	122	68	0.013^*^
	A	98	92	
**rs335929**				
Genotype				
	AA	32	23	0.352
	AC	47	53	
	CC	16	19	
Allele				
	A	123	87	0.767
	C	97	73	
**rs3763043**				
Genotype				
	AA	17	9	0.220
	AG	48	49	
	GG	32	37	
Allele				
	A	92	56	0.179
	G	128	104	
**rs335931**				
Genotype				
	AA	22	19	0.409
	AG	26	20	
	GG	47	56	
Allele				
	A	77	51	0.525
	G	143	109	

* *p < 0.05*.

**Table 5:** Predicted haplotypes from the AQP4 tag SNPs (rs1058424, rs335929, rs376043) between groups.

**Haplotype block**	**rs1058424**	**rs335929**	**rs3763043**	**Frequency**	**χ^2^**	***p*-Value**	**FDR-adjusted *p*-Value (pFDR)**
1	T	A	A	0.127	5.590	0.018^*^	0.144
2	A	C	G	0.101	3.910	0.048^*^	0.192
3	A	A	G	0.159	2.253	0.133	0.356
4	T	C	G	0.312	1.183	0.277	0.553
5	T	A	G	0.039	0.006	0.937	1.071
6	A	A	A	0.228	0.029	0.864	1.153
7	T	C	A	0.023	0.029	0.864	1.153
8	A	C	A	0.012	0.079	0.779	1.246

* *p < 0.05*.

**Figure 2:**
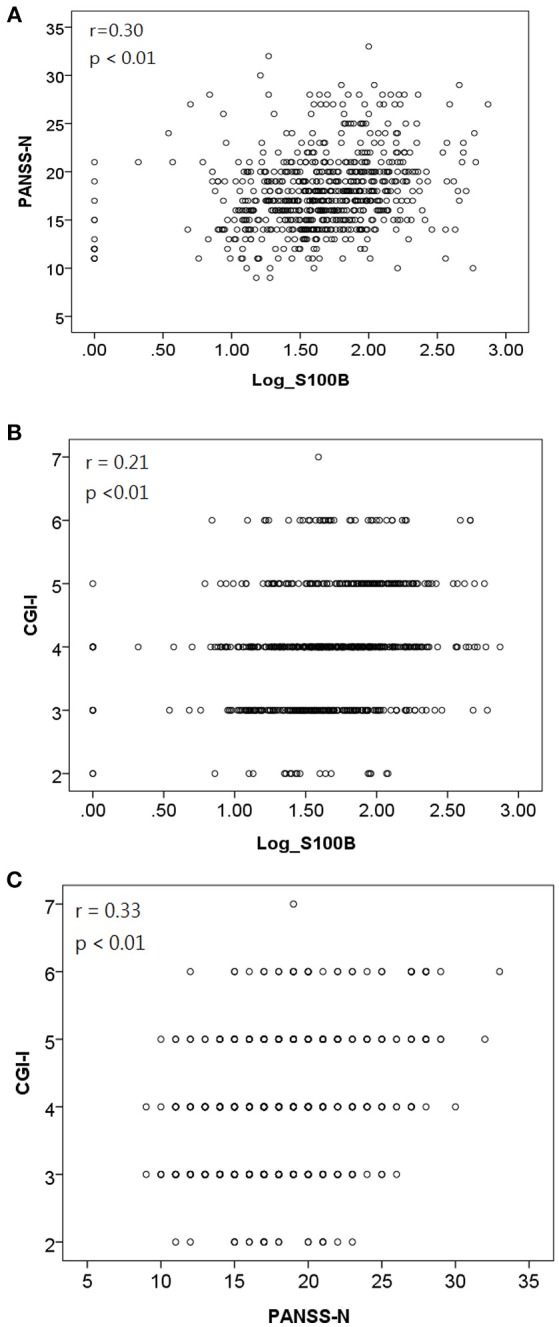
Pearson correlation coefficients indicated a positive correlation between **(A)** log level of S100B and PANSS-N [*r* = 0.30, *p* < 0.01]; **(B)** log level of S100B and CGI-I [*r* = 0.21, *p* < 0.01]; **(C)** PANSS-N and CGI-I [*r* = 0.33, *p* < 0.01].

We used GEE methodology to analyze the data and to investigate the factors possibly related to logS100B level (Table 6). The logS100B levels decreased within the measured time intervals and this achieved statistical significance (β = 1.765, *p* < 0.001) in comparison with the baseline level. The logS100B level had a positive association with the PANSS negative subscale (PANSS-N) score (β = 0.084, *p* = 0.001) and the CGI-I score (β = 0.288, *p* = 0.003). In addition, the TAA haplotype had a positive association with the logS100B level (β = 0.254, *p* = 0.006). Pearson correlation coefficients was proven the positive correlation between log level of S100B, PANSS-N, and CGI-I variables (Figure 2).

**Table 6:** Parsimonious model of changes in variables from the GEE in a 9-week trial.

**Variables**	**β**	**95% Wald CI**	**χ2**	***p*-value**
(Intercept)	1.765	1.706, 1.823	3490.14	< 0.001^***^
Week 9	−0.220	−0.293, −0.148	35.55	< 0.001^***^
Week 6	−0.131	−0.204, −0.057	12.11	0.001^**^
Week 3	−0.143	−0.210, −0.076	17.55	< 0.001^***^
Week 0			
TAA haplotype	0.254	0.072, 0.436	7.49	0.006^**^
PANSS-N	0.084	0.034, 0.133	10.80	0.001^**^
CGI-I	0.288	0.001, 0.480	8.68	0.003^**^

** *p < 0.01*,

*** *p < 0.001, dependent variable, logS100B*.

## Discussion

The activation of glial cells by the brain may represent an effort to fight against neuroinflammation. S100B is a primary product of astrocytes and has been implicated in the regulation of intracellular processes. It exhibits cytokine-like activities and mediates interactions between glial cells and neurons. Increased production of S100B and its release from activated glial cells may act as a cytokine and interfere with neurodegeneration. S100B is a proposed biomarker of SCZ pathophysiology, diagnosis and progression (24). Persistent astrocyte activation, indicated by increased S100B concentration, may be directed toward an ongoing pathogenic process not successfully limited by glial activation. In our study, the high S100B group has significant *P*-value of baseline PANSS and GAF scores maybe due to the persistent influence of neuroinflammation.

The using of antipsychotic monotherapy or polypharmacy with either typical or atypical antipsychotics was allowed according to the severity of illness. As recently summarized, the intervention of antipsychotic drugs can affect glial S100B release (25). Unfortunately, there is no consistent association between S100B level and therapeutic response. Previous studies with repeated measurement have shown either increased or decreased levels of serum S100B during antipsychotic treatment (25). Compared with age- and sex-matched healthy controls, Rothermundt et al. observed patients with SCZ had increased levels of serum S100B both upon admission and after 12 or 24 weeks of treatment (15). However, Ling et al. (26) and Steiner et al. (27) reported that higher baseline levels of S100B in patients with SCZ when compared with levels after 6 or 12 weeks of treatment. Our study had similar findings, showing higher levels at baseline and decreased levels of S100B after 3, 6, and 9 weeks of treatment. In support of our findings, it has been suggested that antipsychotic medication may decrease S100B levels and control the neuroinflammation in patients with SCZ.

Both negative symptoms and function impairment are often enduring and resistant to conventional treatments in individuals with SCZ (28). Previous studies on elevated S100B levels have shown that they are partly correlated with acute exacerbations, compatible with acute illness, and the severity of negative symptoms, compatible with chronic illness (29, 30). Furthermore, persistently high S100B concentrations correlate with memory impairment in patients with chronic SCZ (31). This shows that negative symptoms may significantly contribute to more severe functional disabilities and outcomes (32). In our study, we demonstrated that patients who had a logS100B value >1.78 showed greater severity of negative symptoms.

In contrast to a recent study, we did not observe a strong association between S100B level and the PANSS positive subscale under antipsychotic medication (33). In addition, no significant correlation was observed between S100B and the PANSS total score or its general subscale scores. On the contrary, our study found that the serum S100B level had a positive association with the CGI-I and PANSS-N scores after 9 weeks of treatment. A possible explanation for this may be the influence of age and duration of illness. As shown by the descriptive analysis of demographic characteristics in Table 1, high S100B group had higher average age and longer duration of illness in comparison with low S100B group. Although the CGI-I score improved, the elevated PANSS-N scores seemed to be the core problem in older patients with chronic illness.

Past studies representing an unexpected extent of correlation and structure in haplotype patterns (34, 35) have led to the development of the Human Haplotype Map project (HapMap) and the benefit of genetics research. Our study shows the possible correlation of a rs1058424 polymorphism and a TAA haplotype of the AQP4 gene in the clinical outcome of patients with SCZ. It is therefore possible that the rs1058424 polymorphism within the 3′UTR may regulate the function of the AQP4 gene. This is believed to be the first study to find an association between the haplotypes of the AQP4 gene polymorphisms and S100B activity in a Southern Chinese Han population. We concluded that patients with the TAA haplotype of AQP4 had an increased level of S100B that may be due to persistent neuroinflammation. The expression of the TAA haplotype seemed to be associated with an increased risk of disease progression and poor treatment response. The increase in S100B concentrations appeared to be functionally related to increased negative symptoms of SCZ.

It is important that the expression of the AQP4 gene regulated by genetic polymorphisms may influence blood flow and fluid balance, and consequently the extent of neuroinflammation. The use of specific haplotypes as potential biomarkers is benefit to clarify different subgroups of patients and identify the potential aquaporin modulators in the management of neuroinflammation in SCZ.

Our study had several limitations that are applicable to genetic association studies. We cannot exclude the possibility of selection bias because our participants were selected mainly on the basis of repeated hospital admissions, and cases at the first onset of SCZ may have been under-represented. In addition, we cannot draw conclusions about specific associations between drugs and treatment response because the patients were allowed to receive different types and doses of antipsychotics. We used a graphic method to dichotomize the two groups using selected cutoff points. This approach may in some instances have altered the study outcome when used instead of the traditional analysis in comparison with healthy individuals. Finally, the lack of measurement of inflammatory cytokines provided less powerful evidence of an association between neuroinflammation and AQP4 polymorphisms and haplotypes.

## Conclusions

AQP4 seems to influence brain neuroinflammation in SCZ because of its important role in maintaining BBB integrity, structure, and permeability. Our study provides possible association between the involvement of genetic variations in the AQP4 gene and the functional outcome of patients with SCZ. The risk variants in the AQP4 gene, including the T allele of rs1058424, A allele of rs335929, A allele of rs3763043 and TAA haplotype, are associated with elevated S100B level and greater severity of negative symptoms in individuals with SCZ. The increased interest in AQP4 derives from its potential use as a therapeutic target in patients with SCZ, for the prevention and treatment of negative symptoms, because inhibitors of the TAA haplotype of AQP4 are expected to protect the brain from persistent neuroinflammation. Further study including healthy controls and patients with first and recurrent episodes under selective AQP4 modulators will be necessary to explore the profound effects on the treatment of patients with SCZ and may positively influence their overall outcome.

## Author Contributions

Y-FW, with help of H-KS, and F-WL, planned the present study's content and analysis, interpreted the data and wrote the paper. Y-FW, H-KS, and F-WL initiated and performed the whole survey, analyzed the data and helped to interpret the findings and to write the paper. All authors read and approved the final manuscript.

### Conflict of Interest Statement

The authors declare that the research was conducted in the absence of any commercial or financial relationships that could be construed as a potential conflict of interest.
